# Electrospun Nano-Fibers for Biomedical and Tissue Engineering Applications: A Comprehensive Review

**DOI:** 10.3390/ma13092153

**Published:** 2020-05-06

**Authors:** Shokoh Parham, Anousheh Zargar Kharazi, Hamid Reza Bakhsheshi-Rad, Hamid Ghayour, Ahmad Fauzi Ismail, Hadi Nur, Filippo Berto

**Affiliations:** 1 Biomaterials Nanotechnology and Tissue Engineering Faculty, School of Advanced Medical Technology, Isfahan University of Medical Sciences, Isfahan 8174673461, Iran; shokoparham@gmail.com (S.P.); a_zargar@med.mui.ac.ir (A.Z.K.); 2 Advanced Materials Research Center, Department of Materials Engineering, Najafabad Branch, Islamic Azad University, Najafabad, Iran; hamidghayour70@gmail.com; 3 Advanced Membrane Technology Research Center (AMTEC), Universiti Teknologi Malaysia, Skudai, Johor Bahru, Johor 81310, Malaysia; afauzi@utm.my; 4 Centre for Sustainable Nanomaterials, Ibnu Sina Institute for Scientific and Industrial Research, Universiti Teknologi Malaysia, UTM Skudai, Johor 81310, Malaysia; hadi@kimia.fs.utm.my; 5 Central Laboratory of Minerals and Advanced Materials, Faculty of Mathematics and Natural Science, Universitas Negeri Malang, Malang 65145, Indonesia; 6 Department of Mechanical and Industrial Engineering, Norwegian University of Science and Technology, 7491 Trondheim, Norway

**Keywords:** electrospinning, fabrication, parameters effect, nano-fibers, drug delivery, wound dressing

## Abstract

Pharmaceutical nano-fibers have attracted widespread attention from researchers for reasons such as adaptability of the electro-spinning process and ease of production. As a flexible method for fabricating nano-fibers, electro-spinning is extensively used. An electro-spinning unit is composed of a pump or syringe, a high voltage current supplier, a metal plate collector and a spinneret. Optimization of the attained nano-fibers is undertaken through manipulation of the variables of the process and formulation, including concentration, viscosity, molecular mass, and physical phenomenon, as well as the environmental parameters including temperature and humidity. The nano-fibers achieved by electro-spinning can be utilized for drug loading. The mixing of two or more medicines can be performed via electro-spinning. Facilitation or inhibition of the burst release of a drug can be achieved by the use of the electro-spinning approach. This potential is anticipated to facilitate progression in applications of drug release modification and tissue engineering (TE). The present review aims to focus on electro-spinning, optimization parameters, pharmacological applications, biological characteristics, and in vivo analyses of the electro-spun nano-fibers. Furthermore, current developments and upcoming investigation directions are outlined for the advancement of electro-spun nano-fibers for TE. Moreover, the possible applications, complications and future developments of these nano-fibers are summarized in detail.

## 1. Introduction

As one-dimensional nano materials, nano-fibers have attracted a lot of attention from researchers and found widespread applications [1]. Compared to many other common base materials, nano fibers have numerous important characteristics, including a very thin diameter (a 1000 times thinner than the diameter of human hair), three dimensional topography, flexible surface capabilities, large surface area, adjustable porosity, and good mechanical characteristics such as tensile strength and stiffness [2]. Owing to the progress of nano-fiber technology, an interesting point in this regard is that a large number of different materials can be utilized for the fabrication of these fibers. Such materials include synthetic and natural polymers, metals and their oxides, composite as well as carbon based nano-materials [3]. Additionally, it is possible to modify the surface of nano-fibers together with their bulk properties for different purposes, and this result in a variety of physical qualities and many different applications and usages [4]. The nano-fiber production methods are categorized into two main groups, namely top-down and bottom-up techniques. In a top-down procedure, like mechanical and chemical treatment of wood pulp, nano-fibers are obtained by breaking down the bulk material. Top-down techniques are usually used for obtaining cellulose nano-fibers or CNFs [5]. On the other hand, in bottom-up methods such as electro-spinning, drawing, self-assembly, template synthesis, and phase separation, nano-fibers are fabricated from constituting molecules. Due to their distinctive features that make them quite appropriate for drug delivery systems and biomedical engineering, nano-fibers have been the target of attention of many scientists and researchers [6,7]. These unique characteristics include high porosity, tunable pore size, high surface to volume ratio, as well as morphological correspondence with the extra-cellular matrix. Polymeric fibrous structures were identified as suitable candidates for drug-release systems, and fibrous polymer structures have been prepared by a variety of methods like the electro-hydrodynamic techniques (EHD). In the electro-hydrodynamic methods, electrostatic forces are employed for the production of fibers or particles with an adjustable micro-structure [6]. As an outstanding cost-efficient EHD method, electro-spinning has found vast applications in the industry and laboratory for fiber fabrication. In this method, pharmaceutical biomolecules or agents are directly encapsulated within the fibers, leading to their protection from the environmental parameters and, simultaneously, to the release control [5]. This approach is an extensively employed method compared to other fabrication approaches, due to such properties as low cost, simplicity, and potential of mass production. On top of that, it is feasible to control the composition, orientation and diameter of the nano-fibers in accordance with the desired application [8]. When exploited for the fabrication of wound dressing, electro-spinning offers many benefits, including adjustable porosity, great surface-to-volume ratio, and the capacity of imitating the tissue extra-cellular matrix [3]. As a consequence of these characteristics, on one hand, cytocompatibility will be enhanced, and on the other hand, there will be a high potential of keeping the wound moisture microenvironment and absorbing the wound [9]. According to literature, an electro-spinning setup consists of four basic parts: a glass syringe that contains a polymer solution, a metallic needle, a power supply, and a metallic collector with an adaptable shape [10]. The approach initiates with the motion of electric charges in the polymer solution by means of the metallic needle. Subsequently, the polymer solution becomes instable as the charges are induced on the polymer droplet [11]. Simultaneously, an opposing force to the surface tension is generated by the reciprocal repulsion of the charges. Eventually, the polymer solution flow will be aligned with direction of the electric field. More escalation in the electric field results in the deformation of the droplet shape from spherical to conical. At this point, ultrafine nano-fibers will appear through the conical polymer droplet. These fibers are gathered on the metallic collector that is maintained at an adjusted distance [2]. Essentially, as soon as the polymer solution presented enough cohesive force, a constant charge jet could be created. During the procedure, the liquid jet whipping in the path of the collector will be induced by the inner and outer charge forces [11]. The whipping motion causes the stretching of the polymer chains inside the solution and their sliding past each another, which consequently leads to the formation of fibers that can aptly be called nano-fibers due to their very small diameters [12]. In the recent years, this method has attracted a lot of attention from biomedical researchers from all over the world for fabricating micro- and nano-structures to be applied as drug carriers and scaffolds. In electro-spinning, in order to produce polymeric and non-woven fibers, an electrical field is applied [13]. Three-dimensional scaffolds with different sizes and shapes can be obtained via such a versatile technique. An electro-spinning apparatus setup comprises three main components: a grounded metal collector, a solution reservoir associated with a spinneret, and a high voltage power supply [14]. The properties of the solution and the process parameters are important for the fibers physical, morphological, and biomechanical performance. This cutting-edge technology has a high potential in controlled drug delivery and tissue engineering because an extensive range of materials can be used [15].

**Figure 1:**
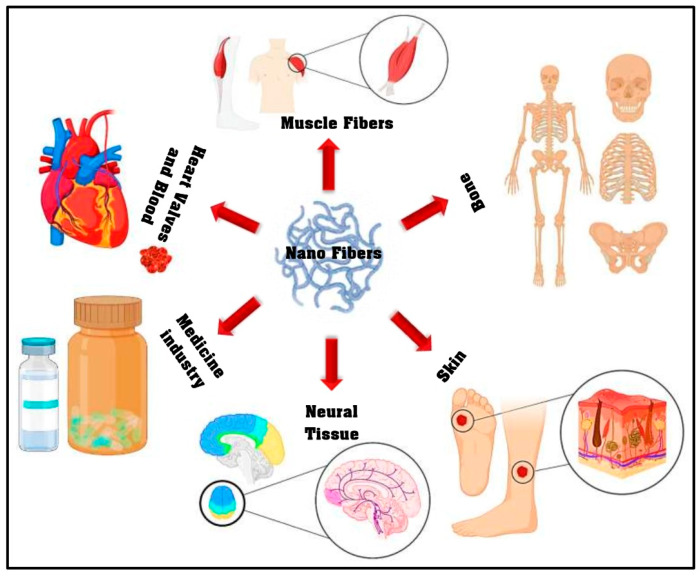
The electro-spun nano-fiber application in different biomedical fields (adapted from Miguel et al. [21]).

There are many different electro-spinning methods such as coaxial, single jet, emulsion, and needleless. Among these, the coaxial electro-spinning or core–shell systems have a multidimensional nature [2]. To illustrate, it is possible to fabricate a continuous double layer of nano fibers with this method. For this purpose, two materials that encapsulate different drug types are electro-spun in a simple one-stage process [16]. On-demand biomaterial platforms for drug delivery and tissue engineering have been provided by this technique through the generation of structures that have two separate parts, an inner part, or core, that is completely enclosed by an outer part, or shell [17,18]. Such a porous structure possesses a morphology that can be beneficial for wound dressing and also drug delivery. In the case of full thickness wounds, since the local vessels have been destroyed, the blood supply encounters obstacles which will inevitably delay the healing of the wound as well as the delivery of the drug [19]. Moreover, wound dressing polymers should be as flexible as the skin and, at the same time, should be capable of loading and releasing the drugs in a controlled mode. Another noteworthy factor that is essential in therapeutic procedures is the reduction of overall dose and this is offered by nano-fibers as they enhance the drugs bioavailability and dissolution [20]. The electro-spun nano-fibers can be exploited for different purposes; in addition to drug delivery systems, tissue engineering, and wound dressing, it has been used in many different fields of biomedicine as can be seen in Figure 1 [21]. Nevertheless, even with the extensive utilization of the electro-spinning approach, the comprehension of this technique is very limited, even now. Hence, the recent review article covers the research carried out on the electro-spinning method, optimization parameters, and pharmaceutical uses of the nano-fibers (range of 50–900 nm) obtained via electro-spinning with the aim of yielding a complete understanding of this approach for tissue engineering (TE) applications. In the subsequent sections of this review article, a comprehensive summery is offered on the cell viability, antimicrobial activity and in vivo analysis of the electro-spun nano-fibers.

## 2. Electro-Spinning for Biomedical Applications

**Figure 2:**
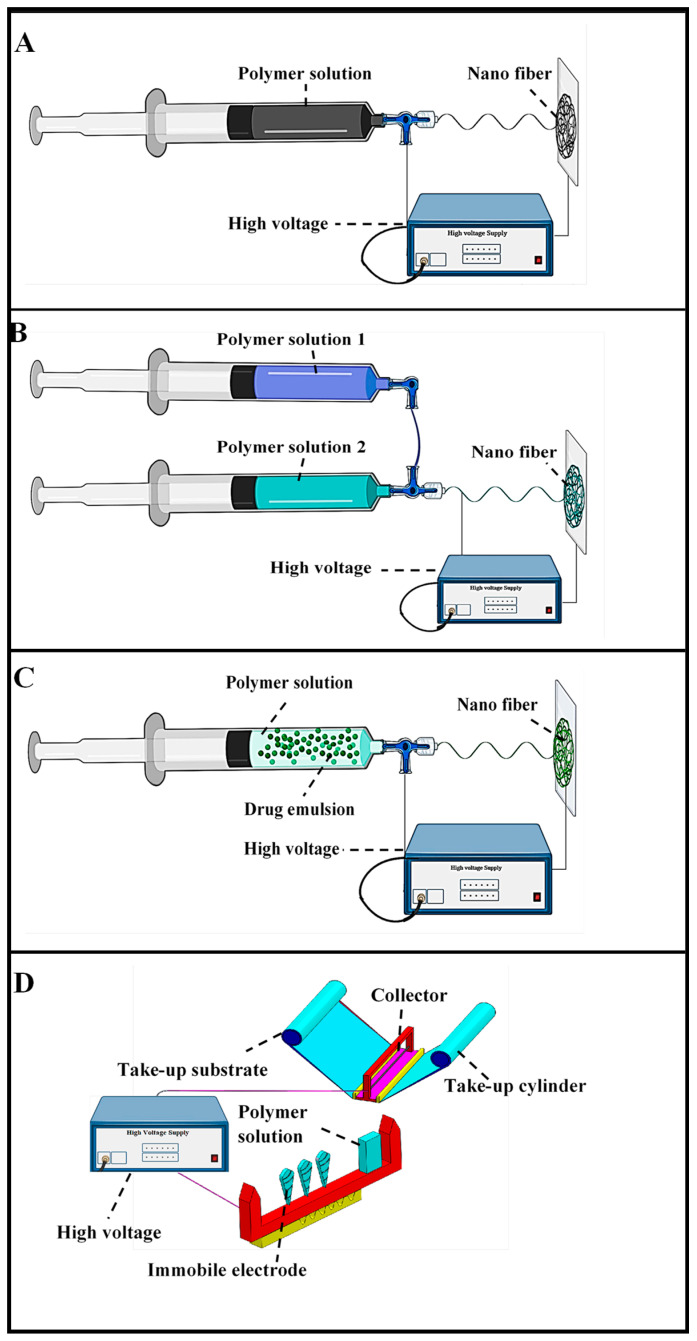
Electro-spinning methodologies (**A**) Blend electro-spinning, (**B**) Coaxial electro-spinning, (**C**) Emulsion electro-spinning, (**D**) Needleless electro-spinning (adapted from Repanas et al. [75], Heydari et al. [92], Manuel et al. [104], and Ambekar et al. [126]).

There is a lot of research investigating antimicrobial biomedical application [22,23,24,25,26,27]. Many researchers have studied the electro-spinning method for biomedical application [28,29,30,31,32,33,34,35,36,37]. Multi-level structured nano-fibers have been successfully developed via electro-spinning for different purposes through the use of such diverse materials as polyacrylic acid, silica nylon, polyacrylonitrile, SU-8 2100, polycarbonate, polybenzimidazole, polyurethane, polyethylene oxide, polystyrene, polyvinyl alcohol, gelatine, cellulose, chitosan, chitin, and carbon nano-materials [36,37,38]. Electro-spun fibers with various shapes such as cylindrical, hollow, porous, ribbon beads, patterned mats, and helices have been developed through the use of different polymers by numerous research teams [39]. The electro-spinning methods include such techniques as blend, coaxial, needleless, and emulsion (Figure 2). Among these, coaxial electro-spinning is used more extensively for biomedicine applications such as wound dressing [21,33]. Due to the production of non-defect nano-fibers, the electro-spinning parameters should be highlighted to attain a significantly better understanding of the electro-spinning approach and the effects of virtually all of these types of regulating parameters. Therefore, Table 1 will help to provide an overview of the parameters of electro-spinning different polymers such as the solvent, tip collector distance, and voltage which have significant effects on the quality of the fabricated nano-fibers [40,41,42,43,44,45,46,47,48,49,50,51,52,53,54]. Table 2 depicts the effects of the electro-spinning equipment setup and their corresponding parameters on the characteristics of polymer-based electro-spinning [55,56,57,58,59,60,61,62,63,64,65,66,67,68,69,70,71,72,73,74]. The fiber morphology is a factor of great significance to guide the application of electro-spun nano-fibers in numerous manufacturing areas. To be able to enhance and/or attain various morphologies, numerous electro-spinning equipment setups and their corresponding experimental parameters including solvent and voltage should be evaluated, since these factors have a significant effect on the cell adhesion and proliferation as well as antibacterial performance of polymers. Hence, these characteristics are summarized in Table 2 with the aim of fulfilling polymer-based electro-spinning applications requirement.

### 2.1. Blend Electro-Spinning

Blend or single jet electro-spinning, as the most common electro-spinning method, involves the mixing of all chemical compounds and polymer components by a single-solvent system. It is possible to encapsulate lipophilic and hydrophilic drugs, as well as biomolecules (like proteins, RNA, and DNA) within the fibers by blend electro-spinning [75,76]. Due to the exceptional characteristics of the fibers, this method is quite effective for the generation of drug delivery systems (DDSs) with sustained diffusion, minimum dosage and local delivery of drugs that will reduce systematic absorption. As a consequence, side effects that result from high dosages can be limited or even avoided. Compared to other methods and DDSs, electro-spun fibers offer reduced initial burst release [77]. Nevertheless, there are some limitations of blend electro-spinning, specifically in terms of drug delivery. For instance, the organic solvents that are used in electro-spinning denature sensitive pharmaceutical proteins, molecules or even DNA. This results in the loss of bioactivity which, in turn, reduces the efficiency of these molecules and proteins [78]. Additionally, due to the electric charge of most bioactive molecules and their ability to migrate to the surface of the fiber (as a result of electrostatic repulsion), distribution within the fibers might be casual [79]. This may ultimately result in the burst release of the encapsulated materials when the fibers are located in an aqueous medium [80]. This technique has been used by many researchers for the improvement of tissue engineering and biomedical applications [78,79,80,81]. To illustrate, bombyx mori silk fibroin nano-fibers obtained by blend electro-spinning were used in wound healing [82]. Poly (L-lactide) + Poly (D-lactide) and Polycaprolactone (PCL) nano-fibers prepared by the same method have also been used for enhancing biomedical application [83,84]. In another study, cellulose acetate (CA) nano-fiber was employed as wound dressing [85]. A simple technique involving a combination of calcination and electro-spinning was exhibited by Kurniawan et al., [86] who fabricated electro-spun poly (N-vinylpyrrolidone) mats that were decorated with gold nano-particles. Due to the electrostatic interactions of the positively charged amino-silane groups with the negatively charged gold nano-particles, the gold nano-particles were decorated on the nano-fibers’ surfaces to improve the antimicrobial activity of the nano-fibers [86]. In blend electro-spinning, the bioactive molecules dispersed or dissolved in the polymeric solution are encapsulated. After that, when the mixture is electro-spun, hybrid fibers will be obtained [82]. As the biomolecules are entrapped within the fibers, the release will be sustained and an early burst release will be prevented [82]. Li et al. prepared an electro-spun nano-fibrous mesh by mixing sodium alginate and organic rectorite (OREC) (which is a bacterial inhibitor) with polyvinyl alcohol (PVA). The in vitro results showed that the nano-fiber’s bactericidal activity was improved by OREC [87]. Chouhan et al. [88] depicted non-mulberry silk fibroin based (NMSF) electro-spun mats functionalized with epidermal growth factor (EGF) and ciprofloxacin HCl as potential wound dressing. Their findings exhibited that the NMS-based mats were cytocompatible, had a high water retention capacity, exhibited antibacterial activity and sustained drug release. Although the blend electro-spinning method is an adaptable and versatile approach, an important drawback of the fibers obtained by this method is the reduction of performance and activity of the embedded biomolecules which accommodates the system’s healing rate [75]. To overcome this limitation, co-axial and emulsion electro-spinning methods have been utilized for fabricating core–shell fibers. The nano-fibers achieved in this manner show an accelerated bioactive/drug molecule encapsulation efficiency and avoid the instantaneous interaction of these biomolecules with the outer media, which is essential for the retention of the biological activity of unstable biological agents [2].

### 2.2. Coaxial Electro-Spinning

Contrary to the blend electro-spinning technique, coaxial electro-spinning is a new variation of the electro-spinning technique which produces multi-layered core-sheath structured nano-fibers (or coaxial fibers) and includes the utilization of two concentrically assembled nozzles linked to an excessive amount of voltage source [89]. The polymeric solution is led through their corresponding reservoirs into the coaxial spinneret by using two individual pumps while controlling the solution flow rate [90]. The mixing of the solvents will be prevented by this setup and the interface of the solutions occurs only at the tip of the spinneret, where the edges of the concentric nozzles end. Numerous research teams have developed and utilized a unique spinneret to facilitate this process [89,90,91]. Fibers with a core–shell structure will be obtained by this setup and these coaxial fibers have a potential to be used as pharmaceutical as well as other sensitive biological molecules carriers, and they are also capable of protecting these biomedical substances from environmental parameters and controlling their release mechanism [92]. Simultaneously, coaxial electro-spinning permits the single-stage co-encapsulation of two or more therapeutic additives in various sections of the coaxial fibers, thus providing us with a multiple or dual drug release system [64]. One of the most interesting advantages of coaxial electro-spinning compared to the blend method is that the former uses two different media, hence minimizing the interaction between the solutions and preserving the internal media, where the sensitive biomolecules or pharmaceutical agents are chiefly distributed, from the environment [92]. Moreover, the core and shell polymer concentrations as well as the ratio of core/sheath solution flow rates are the most significant factors in the coaxial electro-spinning method. Lower flow rates of the inner solution have been designated as more promising for achieving a stable procedure [93]. Both core and shell parts of the fibers can be used for encapsulating therapeutic agents and a proper selection of a drug–polymer–solvent system can significantly improve drug encapsulation efficiency and yield a multiple time-step drug release system in which different drugs are released either simultaneously or one at a time [94]. Another interesting technique involves the utilization of the same core and shell solvent while dissolving the therapeutic agent only in the core solution [95]. In this manner, the distribution of the agent near the fiber core will be restrained and, consequently, the diffusion of the drug in the polymeric matrix will be postponed [94]. PEG/PVA core–shell fibers were used for encapsulating salicylic acid while acetylsalicylic acid (ASA) loaded PCL was electro-spun upon a cylindrical collector to obtain tubular scaffolds for sustained delivery of anti-platelet and anti-inflammatory agents [96]. Maleknia et al. reported that the core–shell polyurethane (PU)/chitosan (Cs) nano-fibers can be used as a potential platform for bioactive scaffolds in tissue engineering. In coaxial electro-spinning, different syringe pumps and coaxial needles are used for the generation of both the core, in which the bioactive constituents are generally encapsulated, and the shell that offers protection and ensures the sustainable release of the loaded molecules [97]. In a study by Maleki et al., the capacity of coaxially electro-spun tetracycline hydrochloride-loaded PLGA core–shell nano-fibers was compared with blend fibers (prepared by using the same materials) for sustained drug release [98]. Other researchers prepared wound dressing by using simvastatin, ciprofloxacin, and other drugs via coaxial electro-spinning. Most researchers confirmed that the core–shell nanostructures exhibited better results in terms of controlled drug release [92,99]. Coaxially electro-spun fibers were employed for encapsulating sensitive growth factors (GFs) in the fiber core. According to the in vivo studies, the GFs sustained release was authenticated via applying polyurethane core–shell fibers, which revealed an activity very similar to that of the fresh GFs [100]. Coaxial electro-spinning has been also utilized for incorporating platelets into the fibers to function as GF reservoirs [101]. Likewise, the loading of nucleic acids with or without extra carriers including plasmids seemed to be created through electro-spinning [102]. Synthetic nano-fibered polymer/DNA composite scaffolds composed mainly of a poly (D,L-lactide)–poly (ethylene glycol) (PLA−PEG) block copolymer and poly(lactide-co-glycolide) (PLGA) random copolymer were used for loading plasmid DNA. Their obtained results pointed out that the DNA released immediately from these scaffolds has the ability of cell attachment and effectively protects the protein b-galactosidase [103].

### 2.3. Emulsion Electro-Spinning

In emulsion electro-spinning, the drug aqueous protein solution is emulsified in a polymer solution. Emulsion electro-spinning can be used to construct core–shell nano-fibers without requiring a specific needle setup [104]. The technique involves chemical separation. An emulsion is created within a single solution and consequently, as the solvent evaporates from the electro-spun fibers, the emulsified droplets are organized into two separate phases [105]. Wang et al. produced biodegradable nano-fibrous polycaprolactone/hyaluronan-epidermal growth factor scaffolds by using emulsion electro-spinning. In vitro examination of human skin keratinocytes (HaCaT) and fibroblasts on these scaffolds exhibited a considerable influence of hyaluronan and epidermal growth factor (EGF) on cell adhesion and growth. Additionally, their results indicated that an elevated reproduction of completely functional skin appeared to be enhanced by the EFG-loaded PCL/hyaluronan scaffolds [106]. In a study by Qi et al. [107], nano-fibers with beads-in-string structures via emulsion electro-spinning from either water-in-oil (W/O) or oil-in-water (O/W) emulsion. Ca-alginate microspheres, that work as reservoirs for hydrophilic drugs, were prepared in a reverse emulsion method and then incorporated into PLLA fibers by electro-spinning. They encapsulated the bovine serum albumin (BSA) into the microspheres and demonstrated that BSA was generated from nano-fibers with lengthy periods of release pattern and less rapid release rates compared with the bare Ca-alginate microspheres [107]. Hu et al. assessed the drug release behavior of small molecule drugs from nano-fibrous scaffolds produced by emulsion electro-spinning of either metoprolol tartrate (MPT) or metformin hydrochloride (MH) with poly (3-hydroxybutyric acid-co-3-hydroxyvaleric acid) (PHBV) or poly (ε-caprolactone) (PCL). According to their results, PCL was demonstrated to be a better drug delivery carrier than PHBV, and MPT-incorporated nano-fibers showed less burst release [108]. Yan et al. evaluated the incorporation of Rhodamine B and BSA into nano-fibers by the emulsion electro-spinning method with controlled drug delivery [109]. In other research projects, smooth core-sheath nano-fibers were fabricated via electro-spinning a W/O emulsion with the aqueous phase consisting of water-soluble drugs solution in water and the oily phase consisting of a chloroform solution of an amphiphilic PEG/PLLA diblock copolymer [110]. Emulsion electro-spinning has been regarded as a promising approach for preparing nano-fibrous materials/scaffolds for GF delivery [111,112].

### 2.4. Needleless Electro-Spinning

In this method, the polymer solution is kept in the tank and a uniform distribution is provided on the electrode through rotation. An electrostatic field is employed for creating fibers without implementing needles [113]. Maver et al. addressed the needleless electro-spinning of carboxymethyl cellulose/polyethylene oxide (CMC/PEO)/plant extract blend aqueous solutions for the purpose of fabricating a cellulose-based wound dressing material that would be appropriate for curing severe wounds. The released study and cell viability tests indicated the promising potential of the product to be used for wound care [114]. Jun-Jye Ng and Pitt Supaphol investigated the production of polymer nano-fiber mats through needleless electro-spinning of poly (caprolactone) (PCL), poly (lactic acid) (PLA), and poly (vinyl alcohol) (PVA) [115]. Wang et al. prepared large scale ultrafine chitosan hybrid nano-fibers containing TiO_2_ and/or Ag nanoparticles by needleless electro-spinning and found that the chitosan hybrid nanofibers showed excellent antibacterial activity. It has been reported that nano-fibers from inorganic polymers have been successfully electro-spun by using mixed solutions of a non-inorganic polymer and an inorganic polymer [116]. The needleless electro-spinning of chitosan nano-fiber has been accomplished using a blend of chitosan with another polymer such as poly (ethylene oxide) (PEO) [117]. The poly (vinyl alcohol) (PVA) and poly vinyl pyrrolidone (PVP) were usually fabricated by needleless electro-spinning method [118,119]. In recent years, researchers have paid more attention to needleless electro-spinning for obtaining antimicrobial wound dressings through the use of antimicrobial inorganic agents like metal and metal oxide nanoparticles [119,120]. On the other hand, these inorganic agents have been reported to generate free radicals through their antimicrobial mechanism, leading to their higher level of toxicity [121,122,123,124,125]. The electro-spinning methodologies of different electrospinning methods (blend electro-spinning, coaxial electro-spinning, emulsion electro-spinning and needleless electro-spinning) are shown in Figure 2 [75,92,104,126].

## 3. Drug Delivery Systems Prepared by Electro-Spinning

A very significant feature of the drug delivery systems (DDSs) is that they might minimize the operations frequency and have a constructive influence on the patient’s conformity [19]. Over the past few years, the drug delivery systems based on electro-spun fibers have received increasing attention due to the unique properties of the fibers [10]. Formulations for transdermal, oral, ocular, and parenteral delivery have been developed by research teams [75,95,99]. GFs and proteins have been successfully encapsulated in the blend and coaxially electro-spun nano-fibers, retaining their bioactivity at the same time [127]. Core–shell fibers composed of PCL in the shell and BSA in the core have been synthesized and by the incorporation of a secondary water-soluble polymer in the shell (PEG), the release kinetics of albumin could be vigorously controlled [128]. Diverse pharmacological agents including analgesics, antibiotics, anti-oxidants, and anti-cancer drugs from natural products have been encapsulated into electro-spun nano-fibers [3,11,19]. Mickova et al. fabricated liposome-loaded core–shell nano-fibers (with PVA as the core and PCL as the shell) as a promising drug delivery system with adequate maintenance of the enzymatic activity of horseradish peroxidase (HRP). Furthermore, it has been reported that the scaffolds induced the mesenchymal stem cells (MSC) proliferation, making them competent biomedical candidates for drug delivery [129]. Aiming to obtain better drug-polymer compatibility and to improve the drug activity, Peng et al. suggested the use of a drug delivery system based on a blend of polymers for the sustained release of paracetamol [130]. PCL/polyurethane (PU) and PCL electro-spun nano-fibers were examined as ketoprofen carriers for local chemotherapy and the results were promising [131]. Doxorubicin hydrochloride, an anti-cancer agent, was loaded within PLLA nano-fibers [132]. Paclitaxel and doxorubicin encapsulated into PLA nano-fibers for anti-cancer treatment exhibited different release profiles [19]. Multi-layered chitosan and hyaluronic acid nano-fibrous scaffolds were employed for delivering paclitaxel, and they proved to be capable of preventing the DU145 prostate cancer cells attachment and proliferation even at low doses [133]. As an anti-coagulant and anti-proliferative pharmaceutical agent, dipyridamole has been used for treatment of cardiovascular diseases [134]. Tubular scaffolds were prepared by electro-spinning biodegradable polyurethane urea (BPU) fibers for the sustained release of dipyridamole [135]. Tensile strains and strengths of the obtained scaffolds were comparable to human coronary artery and the release of dipyridamole over 91 days could reduce human platelet deposition and extend human blood clotting time, indicating that the dipyridamole-loaded BPU scaffolds have a potential to be used as acellular biodegradable small diameter vascular grafts (SDVGs) for vascular replacement [136]. Similar results were observed for the PCL-based coaxially electro-spun fibers for encapsulating dipyridamole fabricated by our research team [135]. Although the utilization of a drug additive including dipyridamole might be considered as lacking novelty, a deeper assessment of therapeutic developments would lead to the inference that the most capable medicines might have already been discovered [137]. With a meticulous design of a drug delivery system, the local delivery of drugs with reduced side effects can enable the use of very powerful pharmaceutical agents that were either overlooked or left out of clinical usage [138]. Ciprofloxacin hydrochloride-loaded electrospun polycarbonate urethane nanofiber prepared for wound dressing showed prolonged drug release [139]. Eudragit L100-55 nano-fibers incorporated with diclofenac sodium (DS) exhibited a pH-dependent drug generation pattern when used for the constant release of the anti-inflammatory agent and presented a great possibility to be employed as oral colon-targeted drug release systems [140]. In addition to drugs, vitamins have been encapsulated within electro-spun nano-fibers as transdermal delivery carriers with increased agent activity, and drug delivery systems for cancer treatment showed improved tumor suppression [141]. Furthermore, mechanical characteristics of the electro-spun nano-fibers were almost similar with those of human skin and cartilage [142]. SiRNA, a bioactive macromolecule that has been used as a therapeutic agent against genetic diseases, was encapsulated into polycaprolactone (PCL) nano-fibers and displayed enhanced cellular uptake [143]. Finally, electro-spun nano-fibers have been scrutinized as anti-HIV drug carriers for preventing HIV infection [144]. One method to protect against virus diffusion is the utilization of microbicides that are capable of eradicating viruses and microbes by using drug delivery systems [145]. Electro-spun c-Cbl-associated protein (CAP)-based nano-fibers loaded with either tenofovir disoproxil fumarate or the reverse-transcriptase inhibitor TMC 125 (anti-HIV agents) were produced and displayed appealing outcomes [146]. Due to their capability of dissolution in basic environment, the CAP nano-fibers could regulate the drug release based on the pH of the vaginal fluids. During sexual intercourse, human semen can raise the vaginal pH levels above 7, which leads to the immediate dissolution of the CAP fibers. The combination of antimicrobial properties of CAP with the anti-HIV drug agents could prevent HIV disease in vitro even in substantially minimal doses [146].

The possibilities of different kinds of electro-spun nano-fibers for DDSs to be employed in prohibition, diagnosis and/or treatment seem to be unrestricted, provided that the many manufacturing obstacles are removed and great clinical information is attained via extreme, great quality, and extensive investigation collaborations are formed between pharmaceutical industry and academic foundations within the area of scientific research [147,148]. Table 3 represents a summary of some of the most important applications of the drugs encapsulated within electro-spun fibers as DDSs [92,139,146,149,150,151,152,153,154,155,156,157,158,159,160,161,162,163,164,165,166,167,168,169,170,171,172,173,174,175,176,177,178,179,180].

## 4. In Vivo Assessment of Electro-Spun Wound Dressing

**Figure 3:**
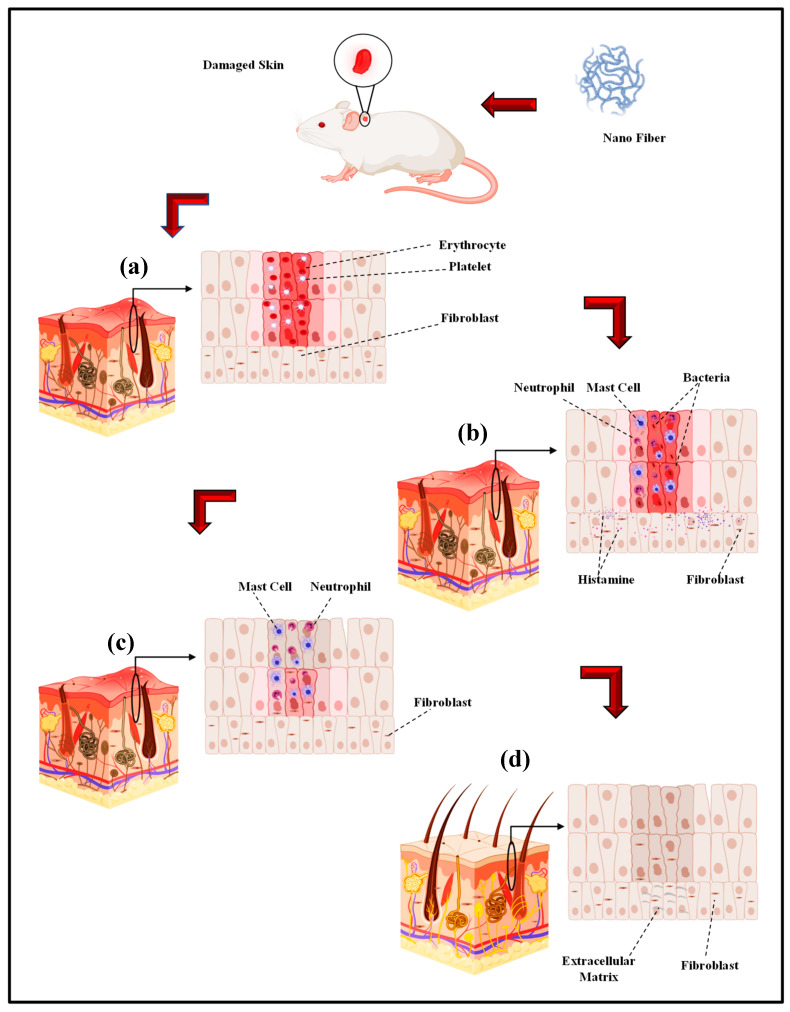
The in vivo wound healing process ((**a**) Hemostasis stage (**b**) Inflammation stage (**c**) Proliferation stage (**d**) Remodeling stage) by electro-spinning wound dressing (adapted from Farokhi et al. [199]).

Electro-spun nano-fibers are mainly fabricated in order to obtain a biomaterial to be used in in vivo tissue regeneration [181,182]. Nonetheless, the first approach to understand the interaction between the cells and substrate and also the materials biocompatibility involves in vitro models. Accordingly, cell cultures are ideal for analyzing a particular cell type in certain circumstances [183]. Consequently, the complexity of the numerous variables of in vivo investigations would be avoided [181,182]. In a study by Ghosal et al., electro-spun nano scale fibers were fabricated by using various polymers and also TiO_2_ composites for wound dressing and tissue engineering. They highlighted the significance of incorporating titanium dioxide nano-particles within or on nano-fibrous scaffolds to impart functional antimicrobial properties, and the relevance of polymer-titanium dioxide nano-composites to wound dressing, drug delivery, and tissue engineering with an emphasis on in vivo biomedical applications [184]. According to Sapru et al., the nonmulberry (Antheraea mylitta) silk protein sericin-based nano-fibrous matrices prepared by electro-spinning displayed enhanced mechanical strength and proper stability (i.e., more than four weeks) as is essential for tissue reconstruction. The sericin-based nano-fibrous matrix also enhanced antibiotic (cephalexin hydrate) delivery [185]. Additionally, they added that antibiotic-loaded nano-fibrous mats accelerated wound healing with negligible inflammation and without any symptoms of illness. The in vitro and in vivo experimentations results, according to Sapru et al., indicate the clear prospect of the prepared nano-fibrous matrices for skin tissue reconstruction [185]. In vivo studies of antibiotic-loaded nano-fibrous matrices presented augmented wound healing with negligible signs of inflammation, regeneration of the lost epidermal layer as well as hair follicles, and neovascularization [99]. Tseng et al. the studies bio-gradable poly [lactic-*co*-glycol acid] (PLGA) nano-fibers for sustainable vancomycin delivery to the brain tissue by using electro-spinning. Their results indicate the great possibilities of these polymeric fibers to effectively address the issues of pharmacokinetics and also pharmacodynamics and to enhance the effectiveness of the therapeutic products that are employed for cerebral infections [186]. By the examination of the in vitro and in vivo release behaviors of pharmaceuticals through the membranes, their experimental outcomes indicated that the high concentrations of vancomycin could be released by bio-gradable nano-fibers pertaining to greater than eight weeks in the cerebral cavity of rats. Additionally, better drug delivery could be achieved without causing negative adverse reactions in the brain. Based on histological examinations, moreover, no inflammation response of the brain was observed, indicating that bio-gradable nano-fibrous drug eluting membranes can be adopted for long term deliveries of different antibiotics in the cerebral cavity [186]. Core–shell polyurethane fibers were used for controlled release of growth factors and, according to in vivo studies, presented an activity comparable to fresh GFs [187]. In another study, hyaluronic acid (HA) nano-fiber was electro-spun and a sterilized HA nano-fiber wound dressing was prepared and compared with four other wound dressings. The in vivo results indicated that the sterilized HA nano-fiber wound dressing was the greatest kind of dressing among other types of dressings [188]. Over the last few years, electro-spun nano-fibers have received the focal attention of many in vivo studies on biomedical applications including wound dressing by different drugs. Electro-spun PVA with phenytoin sodium nano-fibers have been utilized for wound healing purposes and the in vivo results indicated a 90% wound healing in two week [189]. Another researcher reported the PCL nano-fiber embedded with nano-silver in order to improve the wound healing rate and based on the in vivo test results, infection was properly controlled [190]. The PCL nano-fiber with carbon nano-dots (CND) showed in vivo wound recovery and full thickness wound healing in 14 days [191]. PCL Europium hydroxide nano-rods (EHNs) displayed good wound healing properties in the rat model [192]. PCL with ZnO showed 85% wound healing in in vivo situations in 25 days [193]. In the case of CS/PEO nanofibers the 87% wound healing during 10 days and CS/PEO/1wt% cefazolin nano-fibers shows 99% wound healing in the same days [194]. In another in vivo test, the PLGA nano-fiber showed skin tissue regeneration [195]. The PCL/gelatin nano-fibers contain in metronidazole (MNA) demonstrated an in vivo good result in 7 days [196]. PCL/gelatin 6-aminopenicillanic acid (APA) with gold nanoparticles is another electro-spun nano fiber with an outstanding wound healing capacity in in vivo conditions [197]. The polyurethane/keratin/AgNP nanofiber also revealed good in vivo results in wound healing [198]. The in vivo wound healing process: (a) Hemostasis stage (b) Inflammation stage (c) Proliferation stage (d) Remodeling stage by electro-spinning wound dressing as shown in Figure 3 [199].

## 5. Summary and Future Road Maps

Nowadays, nano-fiber technology advances permit the fabrication of nano-fibers from a wide range of materials including natural and synthetic polymers [200,201,202,203,204,205,206,207,208,209,210,211,212,213], ceramics, metals, as well as inorganic/inorganic or organic/inorganic composite systems [214,215,216,217,218,219,220]. A unique combination of properties such as the conductivity of metallic dopants accompanied by the flexibility of polymers can be obtained by a combination of various materials. In this area, the use or incorporation of functional nano-fibers will open new horizons of application for nano-fibrous materials. Presently, many attempts have been made to enhance the nano-fibers properties by developing different fabrication methods. Electro-spinning has the exceptional capability of generating nano-fibers by the use of different materials in different fibrous assemblies. This method has been very interesting for academic and industrial applications as it has a relatively simple setup and a high production rate. Several electrospinning methods have been developed to overcome the main limitations of the standard electrospinning method: low productivity (e.g., needleless electrospinning) and the requirement of organic solvents. These new methods also add new functions, such as controlling nanofiber morphology, compositing, electrospinning of low conductivity polymers and high molecular weight polymers [75].

In brief, electro-spun fibers are competent biomedical candidates, particularly for drug delivery purposes. Electro-spinning is usually effortlessly tuned and employed in a commercial scale to fabricate scaffolds and wound dressings pertaining to clinical purposes. Of course, there is still a lot to understand regarding the features and the usages of the electro-spun nano-fibers. This technology has been found promising in drug delivery and regenerative medicine. While antibiotic drugs and inorganic antimicrobial agents are commonly used for controlling infections, they show more acute side effects like cytotoxicity. Due to toxicity and harmful effects of the previous antibiotic medicines, researchers have been trying to find new antimicrobial agents instead of the previous ones. On the other hand, herbal medicine features antimicrobial and antioxidant antimicrobial activity and low price [221]. Herbal medicine can show positive effects on wound healing [222,223,224]. It is also worth noting that herbal extracts have attracted wide attention, thanks to their affordable cost and minimal negative effects compared to the antibiotic drugs. A blend of herbal extracts with electro-spun nano-fibers is certainly appealing in the field of wound dressing. In particular, these compound extracts have the ability to enhance the wound healing rate and to elevate skin regeneration. Moreover, they have the capability of hydroxyproline formation, cell adhesion, and growth attachment. Therefore, the electro-spun nano-fibers encapsulating herbal medicine have created a new interesting field in all sciences due to their unique properties. These materials can be replaced by antibiotic drugs and other antimicrobial agents like metal and metal oxide nanoparticles in electro-spun nano-fiber wound dressing. The application of these drugs has already resulted in the development of new practical productions. Considering the unquestionable role of bio-application in human life, these new fields in biomedical industry are increasingly welcomed. However, designing a new affordable and applicable system for large-scale production will not only open up a new field of study, but will also meet expanding human needs.

## Figures and Tables

**Table 1:** Parameters of electro-spinning of different polymers.

Polymer	Solvent	Voltage	Tip Collector Distance	Fiber Diameter	Ref.
PCL (Polycaprolactone)	Formic acid/acetic acid	12 kV	12.5 cm	266 nm	[40]
PVA (Polyvinyl alcohol)	Deionized water	22 kV	10 cm	240 nm	[41]
PLA (Poly(lactic acid))	Acetone	20 kV	15 cm	757 nm	[42]
PLLA (poly (l-lactic acid)	Chloroform and acetone	20 kV	12 cm	150 nm	[43]
Chitosan	Acetic acid	40 kV	--	130 nm	[44]
Silk fibroin	CaCl_2_/H_2_O/C_2_H_5_OH	12.5 kV	21 cm	700 nm	[45]
Collagen	Acetic acid	15–20 kV	19–21 cm	100–600 nm	[46]
Hyaluronic acid (HA)	Deionized water	22 kV	15 cm	200 nm	[47]
Poly hydroxyl butyrate	Chloroform: Dichloroethane	20 kV	7.5 cm	280 nm	[48]
Cellulose Acetate	Acetone	12 kV	10 cm	801 nm	[49]
Poly (glycerol sebacate)	Chloroform/ dimethyl formamide	9 kV	30 cm	590 nm	[50]
Elastin	Hexafluoro-2-propanol	10 kV	12 cm	605 nm	[51]
Gelatin	2,2,2-trifluoroethanol (TFE)	10 kV	13 cm	200–300 nm	[52]
Poly (ethylene-co-vinyl alcohol)	Deionized water/isopropyl alcohol (IPA)	15 kV	15 cm	500 nm	[53]
Chitin	1,1,1,2,2,2-hexafluoro-2-propanol (HFIP)	15 kV	7 cm	100 nm	[54]

**Table 2:** Effects of electro-spinning equipment setup and their corresponding parameters on the characteristics of polymer-based electro-spinning.

Polymer	Electrospinning Technique	Fiber Diameter	Application	Condition	Results	Ref.
Collagen + Silk fibroin (SF)	Blend	320–360 nm	Wound healing	In vitro	The cell attachment is 300 cells/0.53 mm^2^	[55]
PCL	Blend	250 nm	Wound healing	In vitro	The duration of the cell culture proliferation is around 7 days	[56]
Poly (L-lactide) + Poly (D-lactide)	Blend	300 nm	Wound healing	In vitro and In vivo	Crystallinity 61% -4 week (Before implantation) and 49% (after implantation)	[57]
Chitosan + PCL	Blend	177 nm	Acute and chronic wound healing	In vitro and In vivo	45% wound recovery in during 6 days	[58]
PCL + BC (Bacterial cellulose)	Blend	400 nm	Wound dressing -	In vitro	The 100% cell viability has been appeared in during 72 h	[59]
Carboxyethyl chitosan (CECS) + PVA	Blend	131–456 nm	Wound dressing	In vitro	The adhesion study of the L929 cells (48 h)	[60]
Chitosan/poly ethylene oxide	Blend	60-120 nm	Wound dressing	In vitro	The viscosity is 2.25 Pa.s and electric conductivity is 3 mS/cm	[61]
PEG-Lysozyme PEG-Bovine Serum Albumin (BSA) + PCL	Co-axial	571 nm	Wound healing	In vitro	50% drug release in during 24 day	[62]
PLA + collagen	Co-axial	168 nm	Wound dressing	In vitro	The cell viability has been increased during 14 days and antimicrobial efficiency against *S. epidermis*, *P. aeruginosa* and *E. coli*	[63]
PLA + Chitosan (CS)	Co-axial	236 nm	Wound healing	In vitro	The antibacterial efficiency against E. coli bacteria	[64]
Chitosan + PEG	Blend	50–200 nm	Wound healing	In vivo	The time of the cells spreading is around 3 days	[65]
Poly-(3-hydroxybutyrate-co-3-hydroxyvalerate) (PHBV)/Ag nanofibers poly	Emulsion	603 nm	Wound healing	In vitro	The drug release of the AgNPs is 0.55 ppm during 30 days	[66]
(Llactide-co-D, L-lactide) + poly (vinyl alcohol)	Blend	275 nm	Wound dressing	In vitro	The anti-bacterial efficiency against *E.coli* and *S.aureus*, the tensile stress is around 19 MPa and Young’s modulus is around 532 MPa and SNL 76/7 fibroblast cell line culture shows good proliferation.	[67]
Dimethyloxalylglycine (DMOG) PCL/Col I	Co-axial	200–500 nm	Wound healing	In vivo	53% drug release of nanofiber during 12 h and 72% during 24 h. drug release of core/shell nanofibers: 17% during 12 h and 36% during 24 h	[68]
Polyhydroxybutyrate (PHB) + Gelatin (GEL)	Blend	80 nm	Wound healing	In vitro and in vivo	The 71.8% degradation rate during 12 h	[69]
Gelatin/Oleoyl Chitosan (OC)	Blend	150–400 nm	Full-Thickness Excisional Wound Healing	In vitro	The swelling is around 380% and the water contact angle is 80°	[70]
Chitosan + PEO	Co-axial	250 nm	Wound healing	In vitro	The tensile strength is 4.0 MPa and porosity is around 84%	[71]
Gelatin + poly-methyl vinyl ether-altmaleic anhydride (PMVE/MA) + nano zinc oxide	Emulsion	500–700 nm	Wound healing	In vivo	99% wound healing during 10 days	[72]
Dimethyloxalylglycine (DMOG) + PLLA	Co-axial	---	Diabetic wound, chronic wound	In vivo	97% wound healing happened during 15 days	[73]
Polyurethanes without dendrimer + Polyurethanes with NO-releasing dendrimer	Co-axial	393 nm	Wound dressing	In vitro	The NO release during 9 h	[74]

**Table 3:** Summary of the drug delivery of different drugs and their biomedical applications.

Drug	Polymer	Electrospinning Method	Application	Ref.
Ascorbylpalmitate	PCL	Blend	Infection treatment	[149]
Amoxicillin	PCL; PLGA	Blend	Infection treatment	[150,151]
Ampicillin	PMMA/nylon; PCL	Blend; Co-axial	Infection treatment	[152,153]
Berberine	Collagen/ZN	Blend	Infection treatment	[154]
Cilostazol	PCL	Blend	Preventing coagulation of blood	[155]
Cefazolin	Gel; PLGA	Blend	Infection treatment	[156,157]
Cefoxitin	PLGA/PEG-b-PLA	Blend	Infection treatment	[158]
Ciprofloxacin	PCNU; PGS/PHB	Blend; Co-axial	Infection treatment	[92,139]
Captopril	PLLA/PLCL/PLGA	Blend	Preventing the complications of high blood pressure	[159]
Doxorubicin	PEG/PLA	Emulsion	Cancer therapy	[160]
Doxycycline	Span 60; PCL; SLS	Emulsion	Infection treatment	[161]
Fusidic acid	PLGA	Blend	Infection treatment	[162]
Gentamycin	CS, PCL	Blend; Co-axial	Infection treatment	[163,164]
Indomethacin	ERS/ES	Blend	Reducing inflammation	[165]
Ketoprofen	PVP	Blend	Reducing inflammation	[166]
Lidocaine	PLLA	Co-axial	Ventricular treatment tachycardia and nerve blocker	[167]
Mefoxin	PDLA/PLLA; PLGA	Blend	Infection treatment	[158,168]
Metronidazole	PCL	Blend	Infection treatment in periodontal diseases	[169]
Mupirocin	PCL	Blend	Infection treatment	[170]
Nifedipine	PLGA	Blend	Prevent the complications of high blood pressure	[171]
Paclitaxel	PLGA	Blend	Cancer therapy	[172]
Rifampicin	PLLA	Blend	Infection treatment	[173]
Resveratrol	PCL	Blend	Inflammation treatment	[174]
Simvastatin	PGS/PHB	Co-axial	Infection treatment	[92]
Salicylic Acid	CS/ZN	Blend	Infection treatment and reducing inflammation	[175]
Streptomycin	PU/CA/Zein	Blend	Infection treatment	[176]
Tetracycline	PCl/CA/Dextran; PLA/PEVA; PVA/CS	Blend	Infection treatment	[177,178,179]
Tenofovir	PVA; CAP	Blend; Needleless	Treatment of viral infections	[146,180]

CAP: cellulose acetate phthalate; PHB: poly (hydroxybutyrate; PLLA: Poly (l-lactic acid); PEVA: poly (ethylene-co-vinyl acetate; CA: Cellulose acetate; CS: Chitosan; PLCL: poly (lactic-co-Ԑ-caprolactone; ERS:Eudragit RS100; ES: Eudragit S100; Gel: Gelatin; PCL: Polycaprolactone; PDLA: Poly-d-lactide; PEG: Poly (ethylene glycol); PLA: Poly (lactic acid); SLS: sodium lauryl salt; PEUU: Poly (ester urethane) urea; PCNU: Polycarbonate urethane; PLGA: Poly Lactic-co-Glycolic Acid; PGS: Poly (glycerol sebacate); PLLCL: Poly (llactic acid)-b-poly (-caprolactone); PMMA: Poly (methyl methacrylate); PU: Polyurethane; PVA: Polyvinyl alcohol; PVP: Polyvinylpyrrolidone; ZN: Zein.
